# External validation of 5A score model for predicting in-hospital mortality among the accidental hypothermia patients: JAAM-Hypothermia study 2018–2019 secondary analysis

**DOI:** 10.1186/s40560-022-00616-5

**Published:** 2022-05-26

**Authors:** Yohei Okada, Tasuku Matsuyama, Kei Hayashida, Shuhei Takauji, Jun Kanda, Shoji Yokobori

**Affiliations:** 1 Japan Association of Acute Medicine Heatstroke and Hypothermia Surveillance Committee, Tokyo, Japan; 2 Department of Preventive Services, Graduate School of Medicine, Kyoto University, ShogoinKawaramachi54, Sakyo, Kyoto, 606-8507 Japan; 3 Department of Emergency and Critical Care Medicine, Japanese Red Cross Society Kyoto Daini Hospital, Kyoto, Japan; 4 Department of Emergency Medicine, Kyoto Prefectural University of Medicine, Kyoto, Japan; 5 Department of Emergency and Critical Care Medicine, Keio University School of Medicine, Tokyo, Japan; 6 Department of Emergency Medicine, North Shore University Hospital, Northwell Health System, Manhasset, NY USA; 7 Department of Emergency Medicine, Asahikawa Medical University Hospital, Asahikawa, Japan; 8 Department of Emergency Medicine, Teikyo University Hospital, Tokyo, Japan; 9 Department of Emergency and Critical Care Medicine, Nippon Medical School, Tokyo, Japan

**Keywords:** 5A score, In-hospital mortality, Accidental hypothermia, Prediction model, External validation

## Abstract

**Background:**

The 5A score including five components “Age, Activities of daily living, Arrest, Acidemia and Albumin” was developed as an easy-to-use screening tool for predicting in-hospital mortality among patients with accidental hypothermia. However, the external validity of the 5A score has not yet been evaluated. We aimed to perform an external validation of the 5A score model.

**Method:**

This secondary analysis of the multicenter, prospective cohort Japanese Association for Acute Medicine-Hypothermia Study (2018–2019), which was conducted at 87 and 89 institutions throughout Japan, collected data from December 2018 to February 2019 and from December 2019 to February 2020. Adult accidental hypothermia patients whose body temperature was 35 °C or less were included in this analysis. The probability of in-hospital mortality was calculated using a logistic regression model of the 5A score. The albumin was not recorded in this database; thus, it was imputed by estimation. Predictions were compared with actual observations to evaluate the calibration of the model. Furthermore, decision-curve analysis was used to evaluate the clinical usefulness.

**Results:**

Of the 1363 patients registered in the database, data of 1139 accidental hypothermia patients were included for analysis. The median [interquartile range] age was 79 [68–87] years, and there were 625 men (54.9%) in the study cohort. The predicted probability and actual observation by risk groups produced the following results: low 7% (5.4–8.6), mild 19.1% (17.4–20.8), moderate 33.2% (29.9–36.5), and high 61.9% (55.9–67.9) predicted risks, and the low 12.4% (60/483), mild 17.7% (59/334), moderate 32.6% (63/193), and high 69% (89/129) observed mortality. These results indicated that the model was well calibrated. Decision-curve analysis visually indicated the clinical utility of the 5A score model.

**Conclusion:**

This study indicated that the 5A score model using estimated albumin value has external validity in a completely different dataset from that used for the 5A model development. The 5A score is potentially helpful to predict the mortality risk and may be one of the valuable information for discussing the treatment strategy with patients and their family members.

**Supplementary Information:**

The online version contains supplementary material available at 10.1186/s40560-022-00616-5.

## Background

Accidental hypothermia is an emergency condition that is defined as an unintentional decrease in the body temperature to less than 35 °C, [1] and is associated with a high mortality risk due to life-threatening arrhythmias and multiorgan injury. Approximately half of patients with accidental hypothermia need intensive care unit (ICU) admission [1–3]. Furthermore, some of the accidental hypothermia patients require invasive rewarming techniques, such as extracorporeal membrane oxygenation for hemodynamic support [1–3]. On the other hand, in a super-aging society such as Japan, most cases of accidental hypothermia occur in residential settings, and the patients are older, frail, and face difficulties in independent living [2–5]. Some patients in such a population might wish the withdrawal of ICU admission and invasive treatment for avoiding undesirable life-sustaining therapy. In the process of considering treatment strategy, if physicians can provide the critical prognostic information to the patients or their family members, they will make the better clinical decisions considering their values, preference and goals of care [6]. Although some predictors or prediction models in patients with accidental hypothermia have been developed, the reproducibility or generalizability of their predictive performance has not been adequately evaluated [7–13]. Therefore, a valid and reliable prediction model that has been evaluated by an external validation study is needed for decision-making based on evidence-based predictions of prognosis.

The 5A score model developed by Okada et al. is an easily applied predictive tool that was derived from a logistic regression model (Table 1 and Additional file 1: eAppendix1) [7]. The 5A score includes five predictors with an initial “A” (Age, Activities of daily living, Arrest, Acidemia, and Albumin) and indicated high predictive performance for in-hospital mortality. However, in order to apply such a prediction model to clinical settings, generalizability should be evaluated through external validation using a totally different dataset from that used for developing the model [14, 15]. Although the 5A score was evaluated in external validation using a split sample from an original cohort [7], this method was insufficient to determine the feasibility of clinical application, and the 5A score has not been evaluated by external validation using a completely different dataset.

**Table 1:** 5A score

Predictor	Score
Age	
60–69	1
70–79	2
≥ 80	3
ADL	
Disturbance	1
Arrest	
Cardiac arrest or SBP ≤ 60 mmHg	2
Acidemia	
pH: 7.2–7.35	1
< 7.2	2
Albumin	
≤ 3 (mg/dl)	1
Sum	/9

ADL: activity of daily living, SBP: systolic blood pressure. pH: pH value of blood gas assessment on arrival at emergency department. Sum score, 0–3 points: low-risk, 4 points: mild, 5 points: moderate, 6–9 points: high-risk. ADL disturbance, the requirement of partial or total assistance for these activities before the accidental hypothermia event in daily activity such as eating, dressing, moving, and taking the bath or shower

This study aimed to externally validate the 5A score model for predicting in-hospital mortality by using a dataset that is independent from the one used for the development of the 5A score.

## Methods

This study was performed according to the reporting guideline in the Transparent Reporting of a Multivariable Prediction Model for Individual Prognosis or Diagnosis statement [16]. This study was approved by the Ethics Review Board of Teikyo University Hospital, Japan (Approval No: 17-090-2) and of each hospital listed in Additional file 1: eAppendix2. The requirement for informed consent was waived because this study is a secondary analysis of anonymized data provided by the Japan Association of Acute Medicine Heatstroke and Hypothermia Surveillance Committee.

### Study setting and design

This study is a secondary analysis of the Japanese Association for Acute Medicine (JAAM)-Hypothermia 2018–2019 database. This national database is derived from a prospective, observational, multicenter cohort study conducted by the JAAM Heatstroke and Hypothermia Surveillance Committee and includes consecutive patients whose body temperature was 35 °C or less when measured at the scene by emergency medical services or at the emergency department (ED). This database recorded the data of accidental hypothermia patients between December 2018 and February 2019, and between December 2019 and February 2020 from 87 and 89 institutions in 2018 and 2019, respectively, participated nationwide in Japan. These data do not include the clinical data which were used to develop the 5A score model. A detailed description of this database has been previously reported and is included in Additional file 1: eAppendix2 [3].

### Participants

This study included adult (age ≥ 18 years) accidental hypothermia patients whose body temperature was 35 °C or less at ED arrival. Patients whose body temperature data were missing or whose temperature was more than 35 °C at the ED were excluded from this study.

### Data collection and outcomes

The following variables were collected as patient characteristics: age, sex, route to hospital, primary cause of accidental hypothermia, settings (indoor or outdoor), lifestyle, activities of daily living (ADL), vital signs, and results of blood test at hospital arrival. Furthermore, information about rewarming method were collected. Definition and details of these variables are provided in Additional file 1: eAppendix3. The outcome of interest was in-hospital mortality.

### Prediction model of interest

The 5A score model is a prediction model of interest and is scored based on five predictors with an initial “A” on arrival at ED (Age, ADL, Arrest or hemodynamically unstable, Acidemia, and Albumin; Table 1) [7]. The formula for calculating the probability of in-hospital mortality is provided in a supplementary file (Additional file 1: eAppendix1). The details of developing process of 5A score are described in a previous study [7]. In short, the 5A score was originally derived using the J-point registry database which was a multicenter retrospective cohort study database that included accidental hypothermia patients between April 1, 2011, and March 31, 2016, and was conducted in 12 hospitals in the urban areas of the Kyoto, Osaka, and Shiga prefectures in Japan. This score was developed by a logistic model for predicting in-hospital mortality using the clinical information from six of the 12 hospitals in the J-point registry and its internal validity was assessed by bootstrapping. Further, it was evaluated for internal–external validation using data of the other six hospitals in the J-point registry which was different from the data used for the model development. In this study, based on the original definition in the original study of the 5A score model [7], ADL disturbance was defined as the requirement of partial or total assistance for daily activities, such as eating, dressing, taking a bath or shower, and using the toilet, before the event. Albumin was not recorded in the JAAM-Hypothermia 2018–2019 Study; thus, we imputed an estimated value for albumin by using a conversion formula (described in the next section and in Additional file 1: eAppendix4).

### Sample size and missing data

With regard to the sample-size estimation, although there are no generally accepted approaches to estimate the sample size for validating prediction models, a previous study suggested that externally validating a prognostic model requires a minimum of 100 events [17]. Therefore, the present study had an adequate sample size with the number of in-hospital death more than 100. For cases with missing data, we described the frequency of the missing data in Additional file 1: eAppendix5. Missing data in the categorical variables of patient characteristics that are not included in the calculation of the 5A score were categorized as “Unknown”; otherwise, missing data were imputed by a multiple imputation technique using the “missForest” R-package [18]. As described above, the serum albumin value was not recorded in the study’s database, and was estimated from the total calcium value based on the previous reports which indicated that the serum albumin value is linearly correlated to the total calcium value [19–23]. Furthermore, sensitivity analysis confirmed the robustness of the results by using different estimating equations and worst-case scenarios. Details of the estimation of the albumin value and sensitivity analysis are described in Additional file 1: eAppendix4)

### Statistical analysis

We described the patients’ characteristics using the median and interquartile range (IQR) for continuous variables and the number and percentages (%) for categorical variables. To evaluate the discriminative performance of the 5A score model, we calculated *C*-statistics with 95% confidence intervals (CI). Moreover, we calculated the probability of in-hospital mortality by using the formula (Additional file 1: eAppendix1) and the mean probability with 95% CI for each value of the 5A score. Furthermore, based on the definition in the original study [7], patients were categorized into risk groups as follows: low-risk, 0–3 points, mild risk, 4 points, moderate risk, 5 points, and high-risk, 6–9 points. To evaluate the calibration performance, we visually compared the observed outcome and mean probability of in-hospital mortality by using a calibration plot with locally weighted scatterplot smoothing by the score and a bar plot by the risk groups. Moreover, we calculated the other performances using the following indices: Nagelkerke *R*^2^ value, calibration slope, intercept, and Brier Score, which are indices of the accuracy of the prediction [14, 24]. Furthermore, we evaluated the clinical utility of the 5A score model by using net-benefit and decision-curve analysis compared to the reference as age, body temperature and “all treatment strategy” [25]. All treatment strategy is a concept of treatment policy in decision-curve analysis, which describes the net-benefit if all the patients are managed as test positive regardless of the prediction [25]. Statistical analyses were performed using R (version 4.1.2).

## Results

### Participant characteristics

**Fig. 1:**
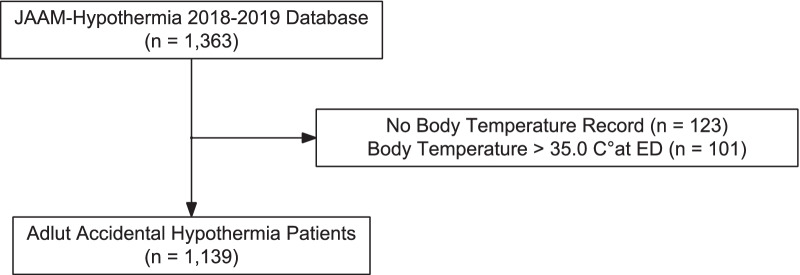
Study flowchart. *ED* emergency department

Among the 1363 patients registered in the database, 1139 patients with accidental hypothermia were included for the analysis (Fig. 1). The median [IQR] age was 79 [68–87] years, 625 (54.9%) participants were male. Almost half of the patients (54%, 610/1139) were admitted to the ICU. In-hospital mortality was reported for 271 (23.8%) participants. Other participant characteristics and in-hospital data are described in Table 2 and Additional file 1: eAppendix 6.

**Table 2:** Patients’ characteristics

Characteristics	*n* = 1139
Age	79.0 (68.0, 87.0)
Sex (men)	625 (55%)
Primary cause	
Alcohol	50 (4.4%)
Disease	566 (50%)
Drowning	31 (2.7%)
Drug	25 (2.2%)
Other	263 (23%)
Outdoor	6 (0.5%)
Trauma	114 (10%)
Unknown	84 (7.4%)
Setting	
Indoor	843 (74%)
Outdoor	263 (23%)
Unknown	33 (2.9%)
ADL disturbance	251 (22%)
Vital signs on arrival	
Cardiac arrest on hospital arrival	101 (8.9%)
BT	30.9 (28.2, 33.4)
SBP	117 (87, 144)
HR	70 (50, 88)
GCS	10.5 (7.0, 13.0)
Blood test result	
pH	7.29 (7.18, 7.35)
Alb (estimated)	3.54 (3.23, 3.77)
Rewarming procedure	
Blanket	419 (37%)
Forced warm air	596 (52%)
Heating pad	12 (1.1%)
VAECMO	35 (3.1%)
HD/CHDF	10 (0.9%)
Intravascular catheter	19 (1.7%)

Continuous variables are described as median and interquartile range and categorical variables are number and percentage

*BT* body temperature, *SBP* systolic blood pressure, *HR* heart rate, *GCS* Glasgow Coma Scale, *Alb* serum albumin, *VAECMO* veno-arterial extracorporeal membrane oxygenation, *HD* hemodialysis, *CHDF* continuous hemodialysis and filtration

### Model performance

**Fig. 2:**
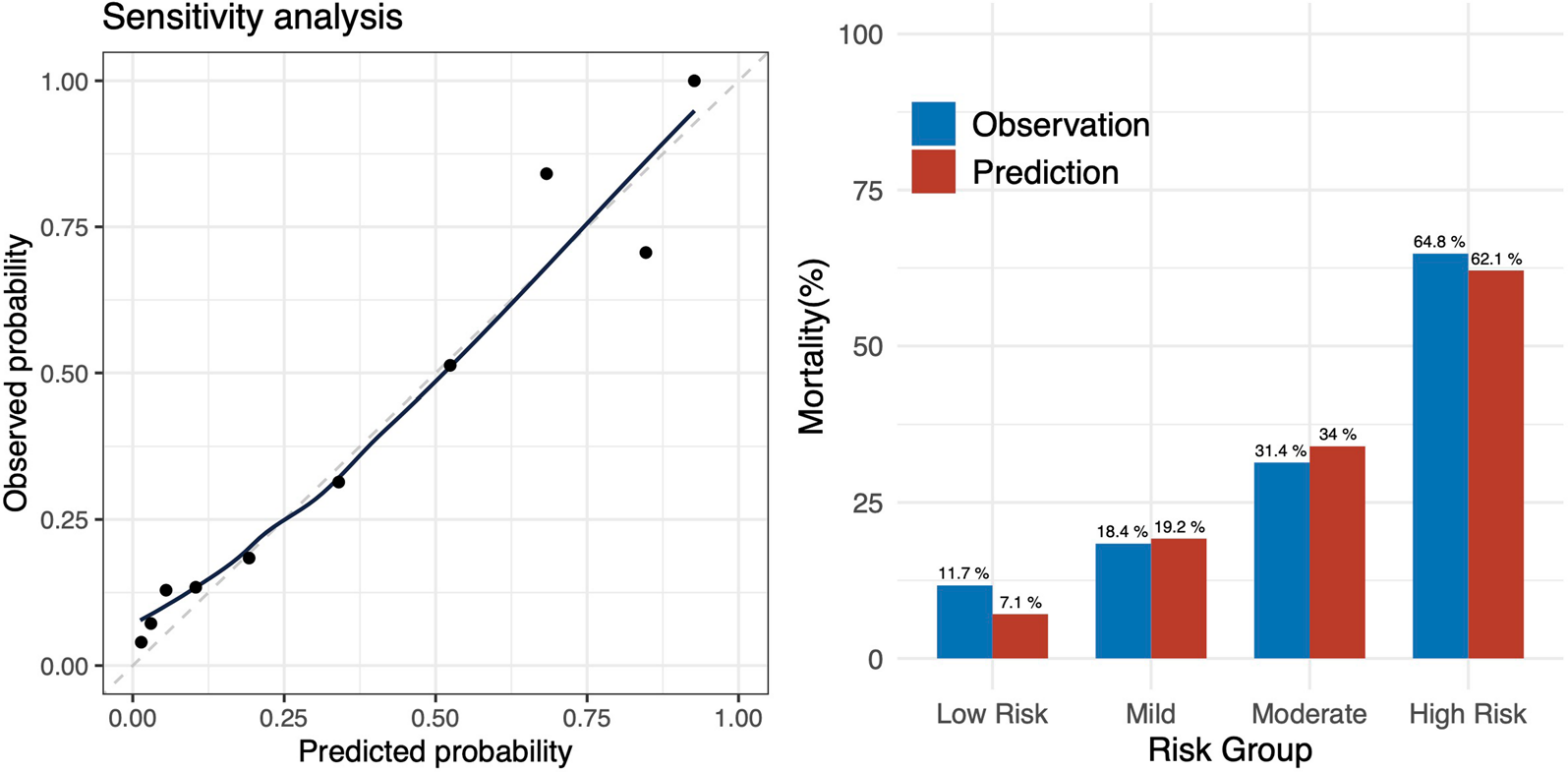
Calibration plot. Left: *X*-axis: predicted probability, *Y*-axis: observed probability, Circle: each score. Right: predicted and observed probability for in-hospital mortality by groups. Low risk: 0–3 points, mild: 4 points, moderate: 5 points, high risk: 6–9 points

**Fig. 3:**
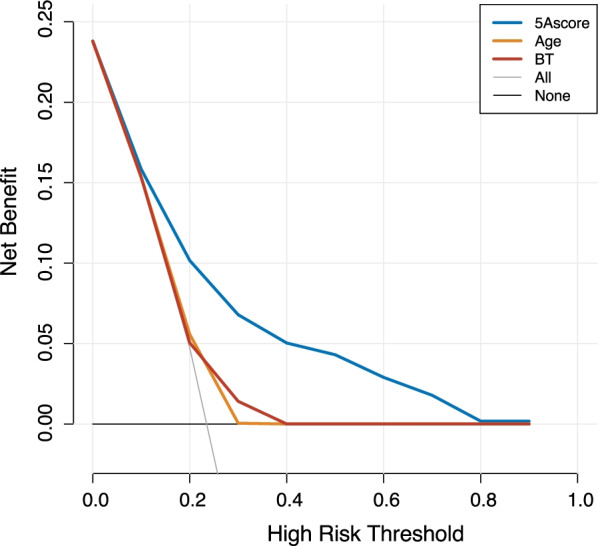
Decision-curve analysis. *BT* body temperature. The net-benefit of 5A score is higher than age, BT, “all treatment strategy” and “none” in almost all range of threshold probability. All treatment strategy is a concept of treatment policy in decision-curve analysis, which describes the net-benefit if all the patients are managed as test positive regardless of the prediction. None is also a treatment policy which describes the net-benefit if all the patients are managed as test negative regardless of the prediction

To evaluate the discriminative performance of the 5A score using the estimated albumin value, we found that the *C*-statistic [95% CI] was 0.736 [0.699–0.772]. For calibration, the predicted probability and actual observation by score are illustrated (Fig. 2, left). Furthermore, the predicted probability [95% CI] and actual observation by risk groups were as follows: Predicted probability, low risk 7% (5.4–8.6), mild 19.1% (17.4–20.8), moderate 33.2% (29.9–36.5), and high risk 61.9% (55.9–67.9); observation, low risk 12.4% (60/483), mild 17.7% (59/334), moderate 32.6% (63/193), and high risk 69% (89/129), as shown in Fig. 2 (right panel). Although the prediction was slightly underestimated in the patients in the low-risk and high-risk groups, the model was well calibrated. Other details of model performance are specified in Additional file 1: eAppendix7, 8. In the decision-curve analysis (Fig. 3), the net-benefit of 5A score model is visually higher than that of age, BT, or all treatment strategy, which suggests the clinical utility of the 5A score model. In the sensitivity analysis, the robustness of the results was confirmed, which is described in Additional file 1: eAppendix9 and 10.

## Discussion

### Key findings

This multicenter observational study conducted the external validation of the 5A score for predicting the in-hospital mortality for the patients with accidental hypothermia. The results indicated that the 5A score using the estimated albumin value is well calibrated and has clinical utility. Thus, the 5A score could facilitate decision-making regarding the treatment strategy.

### Strengths

The 5A score has several strengths. First, this 5A score is the first externally validated prediction model for use in patients with accidental hypothermia. Previously, some reports have indicated methods to predict the clinical outcome for the patients with accidental hypothermia. For example, the “ICE score” and the “HOPE score” were reported for predicting the prognosis of patients with cardiac arrest following accidental hypothermia [11, 12]. However, these scores were developed on the basis of a literature review of case reports and was affected by both publication and selection bias. Furthermore, another prediction model for accidental hypothermia using coagulopathy and the level of consciousness was reported as a prediction tool, but was derived from a small sample (192 patients with accidental hypothermia) and had a risk of overfitting and less reproducibility [9]. Moreover, these scores were not evaluated using bootstrapping for internal validation or with a separate dataset for external validation; therefore, there are serious concerns with regard to the applicability of the model in the real-world clinical settings. However, with regard to the 5A score, internal validation by a bootstrapping procedure and both internal–external validation have already been evaluated previously [7]. Moreover, this study undertook external validation using a completely different cohort from that used for the derivation data. Therefore, the predictive performance of the 5A score may have adequate reproducibility and generalizability to similar populations.

Second, the 5A score constitutes a simple and easy system that may be helpful to rapidly predict the in-hospital mortality in the ED. For critically ill patients, some scores to evaluate multiorgan injury such as the Sequential Organ Failure Assessment Score (SOFA) and Acute Physiology and Chronic Health Evaluation (APACHE) 2 scores are widely accepted and are used to assess the severity. Especially, the SOFA score was utilized in the assessment of patients with accidental hypothermia [8, 10]. However, these scores comprise multiple factors of organ injury as well as many predictive variables, and some of these parameters are unavailable in the ED after hospital arrival. Thus, the above-mentioned scores may be unsuitable for predicting mortality in the ED. Furthermore, when using APACHE2 score or other risk-assessment tools, the predicted mortality cannot be calculated without using a complicated equation. In contrast, physicians in the ED can easily and rapidly use the 5A score to predict mortality.

Third, this study suggested the clinical utility of the 5A score model using decision-curve analysis. The clinical utility of the prediction model is described as the net-benefit, which is the difference between the number of correctly predicted cases and wrongly predicted cases, weighted by the values [27]. For evaluating the prediction model, not only predictive performance, but also clinical utility should be assessed [28]. However, the clinical utility has not been evaluated for the previously reported prediction models used for accidental hypothermia [11, 12, 26]. In contrast, in this study, we evaluated the clinical utility using the decision-curve analysis, which indicated that the 5A score may have a higher net-benefit than BT and age. Based on these strong points, we believe that the 5A score model is an easy-to-use valuable tool in the ED for predicting mortality and facilitating decision-making, with high reproducibility and generalizability.

### Clinical implication

We believe that the 5A score can enable clinicians to rapidly predict the in-hospital mortality risk of patients with accidental hypothermia, and thus provide patients and their families with information about their prognosis to enable the selection of the appropriate treatment strategy based on their values. In urban areas, most accidental hypothermia patients are older adults, and we do not think that all of the patients would want to avail invasive and aggressive life-sustaining treatment. If there is no established prognosis prediction model, physicians would be apprehensive about their therapeutic decision-making with regard to the treatment. Furthermore, in some cases, the above-mentioned deficit might lead to an unfavorable situation wherein an older adult patient who is facing imminent death might be treated too invasively, or patients with good survival prospects might undergo early withdrawal of treatment. If an option for the prediction of the prognosis is available, this can be valuable for discussing the treatment strategy with the patients or their family. For example, for patients in the low-risk group (≤ 3 points) who are expected to survive, aggressive treatment might be acceptable even if the patient is an older adult. On the other hand, for individuals in the severe-risk group (≥ 6 points), prediction of prognosis can be an important information to discuss about treatment strategy with the patient’s relatives. Therefore, the 5A score can provide valuable information to facilitate rational decision-making considering preference and values.

## Limitation

This study had several limitations. First, the serum albumin value, a component of the 5A score, was not measured in this database and it was imputed by estimated value. Even though the robustness of the main results was confirmed through the sensitivity analysis and weight of albumin value in 5A score was not so high (one-nineth), the lack of an objectively measured albumin level is the most important limitation of this study; thus, it should be considered while interpreting the results. Second, although clinical information was prospectively collected in this database, inaccurate data collection or missing data might confer a risk of bias. Especially, in some of the patients, body temperature was measured at the axilla, and it might not exactly reflect the core body temperature in some circumstances. Further, assessing the ADL was not completely objective and it might also contribute towards the risk of a measurement bias. Third, the treatment for the patients was decided by the physician in charge and it may be based on the clinical information including age, ADL and so on, which might be a self-fulfilling prophecy bias. Fourth, the prediction was slightly underestimated among patients in the low-risk and high-risk groups, which may have been caused by overfitting of the model. Fifth, this database was compiled from data derived from more than 89 hospitals nationwide, and generalizability of the results to Japan may be confirmed; however, it is unclear whether this model would be applicable to other settings and in other countries. Lastly, the extent to which this score clinically influences the treatment strategy and actual patient outcomes remains unclear. Especially, it is not appropriate to jump to conclusion of withdrawal of intensive care only based on the 5A score, because even for the patients in the high-risk group, some of the patients (31%) were able to obtain survival discharge in this study. We believe that treatment strategy should be decided comprehensively, not only based on the 5A score, but also based on other clinical information, medical resources, and values of the patients. To eliminate such limitations, further research or updating the model would be necessary.

## Conclusion

In this study, the 5A score model using estimated albumin value was externally validated in a completely independent dataset from that used for the model development. The 5A score is potentially helpful to predict the mortality risk and may be one of the valuable information for discussing the treatment strategy with patients and their family members.

## Supplementary Information

**Additional file 1: eAppendix1.** 5A score model. **eAppendix2.** List of hospitals participating in the database. **eAppendix3.** Detail of the variables. **eAppendix4.** Estimation of the Albumin value and sensitivity analysis. **eAppendix5.** Missing of the variables. **eAppendix6.** Other details of patient characteristics. **eAppendix7. **Performance of 5A score model. **eAppendix8.** Predicted and Observed probability. **eAppendix9.** Results of sensitivity Analysis. **eAppendix10.** Results of Sensitivity Analysis using worst-case scenario.

## Data Availability

The datasets generated and/or analyzed during the current study are not publicly available because it is not approved by the Ethics Committee. Editors are available from the corresponding author on reasonable request.

## Abbreviations

ED: Emergency department
BT: Body temperature
ADL: Activities of daily living
JAAM: Japanese Association for Acute Medicine
SOFA score: Sequential Organ Failure Assessment Score
APACHE2 score: Acute Physiology and Chronic Health Evaluation 2 Scores
