# Correction: External validation of 5A score model for predicting in-hospital mortality among the accidental hypothermia patients: JAAM-Hypothermia study 2018–2019 secondary analysis

**DOI:** 10.1186/s40560-022-00617-4

**Published:** 2022-06-09

**Authors:** Yohei Okada, Tasuku Matsuyama, Kei Hayashida, Shuhei Takauji, Jun Kanda, Shoji Yokobori

**Affiliations:** 1 Japan Association of Acute Medicine Heatstroke and Hypothermia Surveillance Committee, Tokyo, Japan; 2 Department of Preventive Services, Graduate School of Medicine, Kyoto University, ShogoinKawaramachi54, Sakyo, Kyoto, 606-8507 Japan; 3 Department of Emergency and Critical Care Medicine, Japanese Red Cross Society Kyoto Daini Hospital, Kyoto, Japan; 4 Department of Emergency Medicine, Kyoto Prefectural University of Medicine, Kyoto, Japan; 5 Department of Emergency and Critical Care Medicine, Keio University School of Medicine, Tokyo, Japan; 6 Department of Emergency Medicine, North Shore University Hospital, Northwell Health System, Manhasset, NY USA; 7 Department of Emergency Medicine, Asahikawa Medical University Hospital, Asahikawa, Japan; 8 Department of Emergency Medicine, Teikyo University Hospital, Tokyo, Japan; 9 Department of Emergency and Critical Care Medicine, Nippon Medical School, Tokyo, Japan

## Correction to: Journal of Intensive Care (2022) 10:1-8 https://doi.org/10.1186/s40560-022-00616-5

Following the publication of the original article [1], it was noted that due to a typesetting error the Fig. 2 was not the updated version. The correct figure is given below. The original article [1] has been updated.

**Fig. 2:**
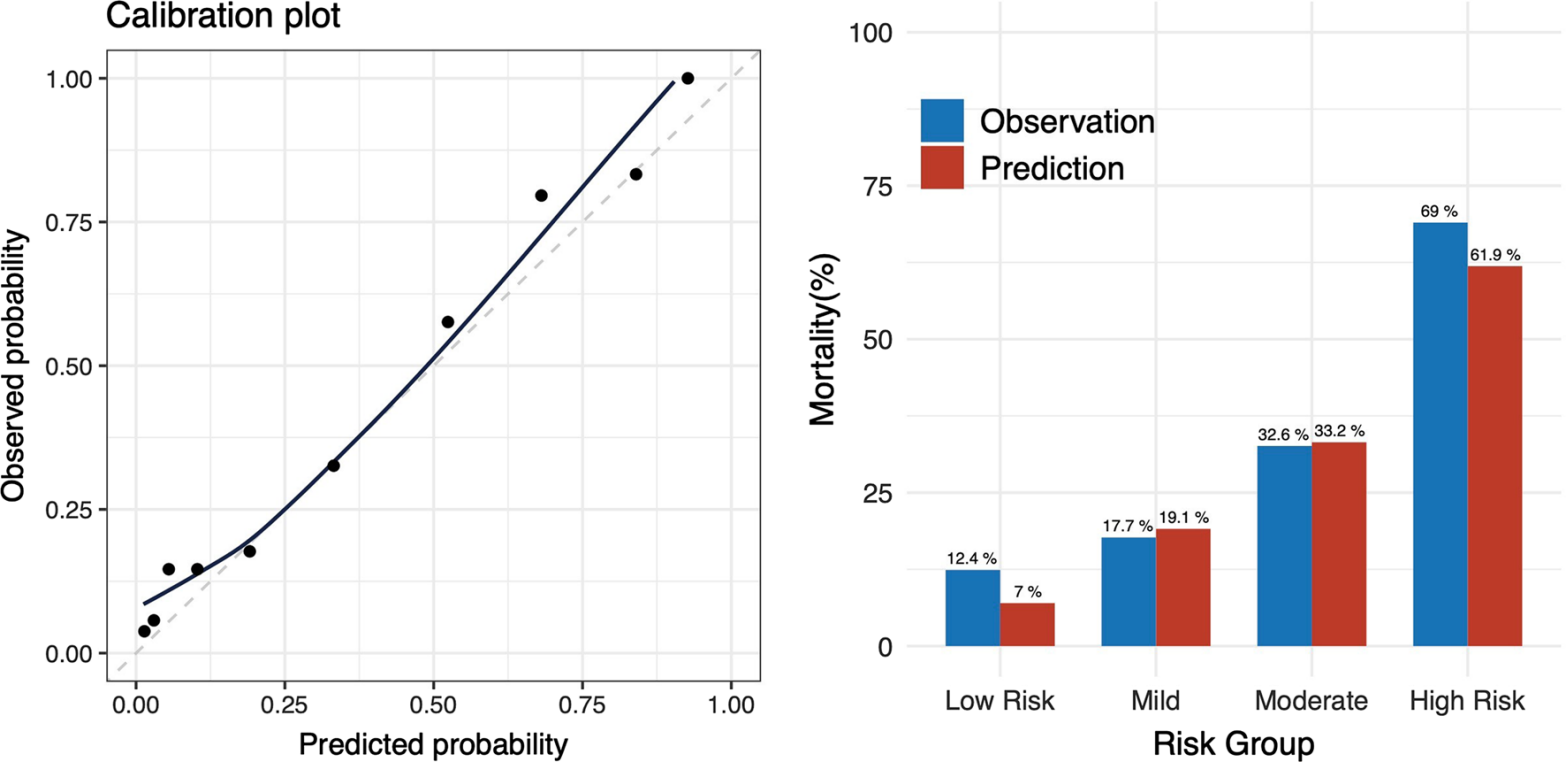
Calibration plot. Left: X-axis: predicted probability, Y-axis: observed probability, Circle: each score. Right: predicted and observed probability for in-hospital mortality by groups. Low risk: 0–3 points, mild: 4 points, moderate: 5 points, high risk: 6–9 points
