# Supplementary material for: External validation of 5A score model for predicting in-hospital mortality among the accidental hypothermia patients: JAAM-Hypothermia study 2018–2019 secondary analysis
Source: J Intensive Care. 2022 May 26;10:24. doi: 10.1186/s40560-022-00616-5 (PMC9134674; doi:10.1186/s40560-022-00616-5)
Supplement: Supplementary file 1 — Additional file 1: eAppendix1. 5A score model. eAppendix2. List of hospitals participating in the database. eAppendix3. Detail of the variables. eAppendix4. Estimation of the Albumin value and sensitivity analysis. eAppendix5. Missing of the variables. eAppendix6. Other details of patient characteristics. eAppendix7. Performance of 5A score model. eAppendix8. Predicted and Observed probability. eAppendix9. Results of sensitivity Analysis. eAppendix10. Results of Sensitivity Analysis using worst-case scenario. [file 40560_2022_616_MOESM1_ESM.docx]

**Additional file**

**Title:** External validation of 5Ascore model for predicting in-hospital mortality among the accidental hypothermia patients; JAAM-Hypothermia study 2018-2019 secondary analysis

**Authors:**

Yohei Okada, Tasuku Matsuyama, Kei Hayashida, Shuhei Takauji, Jun Kanda, Shoji Yokobori

**Contents**

| **eAppendix1.** | **5A score model** |
| --- | --- |
| **eAppendix2.** | **List of hospitals participating in the database** |
| **eAppendix3.** | **Detail of the variables** |
| **eAppendix4.** | **Estimation of the Albumin value and sensitivity analysis** |
| **eAppendix5.** | **Missing of the variables** |
| **eAppendix6.** | **Other details of patient characteristics** |
| **eAppendix7.** | **Performance of 5A score model** |
| **eAppendix8.** | **Predicted and Observed probability** |
| **eAppendix9.** | **Results of sensitivity Analysis** |
| **eAppendix10.** | **Results of Sensitivity Analysis usin**g **worst case scenario** |
| **Reference** |  |

**eAppendix1. 5A score model**

**5A score**

| **Predictor** |  | **Score** |
| --- | --- | --- |
| Age | 60-69 | 1 |
|  | 70-79 | 2 |
|  | ≥ 80 | 3 |
| ADL | Disturbance | 1 |
| Arrest | Arrest or  SBP≤ 60 mmHg | 2 |
| Acidemia | pH: 7.2-7.35 | 1 |
|  | <7.2 | 2 |
| Albumin | ≤ 3 (mg/dl) | 1 |
| **Sum** |  | **/9** |

**Formula for calculating the in-hospital mortality**[1]

Predicted in-hospital mortality = 1/{1+Exp-(ax+b)}

ax+b= - 4.2338 (Intercept)

+Age(60-69y.o)*0.675+Age(70-79y.o)*1.376+Age(≥80 y.o)*1.976

+ADL(distrubance)*0.918

+ SBP(61-90mmHg)*(-0.146)+near-Arrest*1.716

+ pH(7.2-7.35)*0.786+pH(<7.2)*1.217+pH(Unknown)*(-0.006)

+ Alb(≤3 mg/dl)*0.952+Alb(Unknown)*0.146

In original model, unknown category was used, but in this study, missing value was imputed by missForest; thus, unknown category was not used. Disturbance is assessed as the requirement of partial or total assistance for these activities before the accidental hypothermia event, based on the judgement of the physicians or nurses in charge or their families.

**eAppendix2. List of hospitals participating in the database**

Aidu Chuo Hospital

Aizawa Hospital

Akita Red Cross Hospital

Aomori Prefectural Central Hospital

Asahikawa City Hospital

Asahikawa Medical University Hospital

Asahikawa Red Cross Hospital

Center Hospital of the National Center for Global Health and Medicine

Chiba Emergency Medical Center

Chikamori Hospital

Daiyukai General Hospital

Dokkyo Medical University Nikko Medical Center

Dokkyo Medical University Saitama Medical Center

Eastern Chiba Medical Center

Ehime Prefectural Niihama Hospital

Esashi Hospital

Fujieda Municipal General Hospital

Fujisawa City Hospital

Fukui Prefectural Hospital

Fukuoka University Hospital

Fukushima Medical University Hospital

Funabashi Municipal Medical Center

Gifu Prefectural General Medical Center

Gifu University Hospital

Hachinohe City Hospital

Hamamatsu Medical Center

Hidaka Tokushukai Hospital

Hiroshima Prefectural Hospital

Hokkaido Medical Center

Hyogo Emergency Medical Center

Hyogo Prefectural Nishinomiya Hospital

Ina Central Hospital

Ise Red Cross Hospital

Ishikawa Prefectural Central Hospital

Ishinomaki Red Cross Hospital

Iwata City Hospital

Iwate Prefectural Central Hospital

JA Onomichi General Hospital

Japanese Red Cross Society Kyoto Daiichi Hospital

Jichi Medical University Saitama Center

Jikei University Daisan Hospital

Juntendo University Nerima Hospital

Juntendo University Urayasu Hospital

Kagawa University Hospital

Kansai Medical University Hospital

Kasugai Municipal Hospital

Kawaguchi Municipal Medical Center

Kawasaki Municipal Hospital

Kimitsu Chuo Hospital

Kishiwada Tokushukai Hospital

Kitakyushu General Hospital

Kumamoto Red Cross Hospital

Kushiro City General Hospital

Kyorin University Hospital

Kyoto University Hospital

Maebashi Red Cross Hospital

Mie Prefectural General Medical Center

Mie University Hospital

Miyazaki Prefectural Nobeoka Hospital

Nagano Red Cross Hospital

Nagasaki University Hospital

Nagoya Ekisaikai Hospital

Nagoya University Hospital

Narita Red Cross Hospital

Nasu Red Cross Hospital

National Defense Medical College Hospital

National Hospital Organization Mito Medical Center

National Hospital Organization Nagoya Medical Center

National Hospital Organization Osaka National Hospital

National Hospital Organization Yokohama Medical Center

Nayoro City General Hospital

Nihon University Hospital

Nihon University Itabashi Hospital

Nihonkai General Hospital

Niigata University Medical & Dental Hospital

Nippon Medical School Hospital

Nippon Medical School Tamanagayama Hospital

Oita University Hospital

Okinawa Prefectural Nanbu Medical Center & Children's Medical Center

Okitama Public General Hospital

Ome Municipal Central Hospital

Omihachiman Community Medical Center

Osaka City General Hospital

Ota Memorial Hospital

Rinku General Medical Center

Saiseikai Shiga Hospital

Saiseikai Utsunomiya Hospital

Sapporo City General Hospital

Sapporo Medical University Hospital

Seirei Hamamatsu General Hospital

Seirei Mikatahara General Hospital

Shinshu University Hospital

Shizuoka Red Cross Hospital

Shonan Kamakura General Hospital

St.Mary's Hospital

Steel Memorial Hirohata Hospital

Sunagawa City Medical Center

Takasaki General Medical Center

Teikyo University Hospital

Teine Keijinkai Hospital

Tenshi Hospital

Toho University Omori Medical Center

Tohoku University Hospital

Tokai University Hospital

Tokushima Prefectural Miyoshi Hospital

Tokuyama Central Hospital

Tokyo Metropolitan Tama Medical Center

Tosei General Hospital

Toyama University Hospital

Tsuyama Chuo Hospital

Uji Tokushukai Medical Center

University of Tokyo Hospital

University of Yamanashi Hospital

Wakayama Red Cross Medical Center

Yamagata Prefectural Central Hospital

Yamagata University Hospital

Yamaguchi University Hospital

Ymanashi Prefectural Central Hospital

Yokkaichi Municipal Hospital

Yokohama Minami Kyosai Hospital

**eAppendix3. The detail of variables**

| Variable | Description |
| --- | --- |
| Patient characteristics | |
| Sex | Sex (Men/Women) |
| Age | Age |
| Route to hospital | It is categorized from by ambulance, walk-in, by helicopter, others (e.g., transferred from other hospital). If missing, it is categorized as Unknown. |
| Primary Cause | The most likely cause of hypothermia is selected from disease from disease, alcohol related, drug related, drowning, outdoor activity, trauma, other, or unknown.  “Disease” is defined as internal cause such as stroke, hypoglycemia or infection. “Drowning” is drowning or immersion of water. “Alcohol related” is drunkenness. “Drug related” is related drug intoxication such as overdose for attempting commit suicide. “Outdoor” includes avalanche or lost in outdoor activity such as climbing. “Trauma” is related to fall or external event such as accident. If missing, it is categorized as Unknown. |
| Settings | Indoor or outdoor |
| Lifestyle | It is categorized as living alone, nursing home, or homeless, if applicable, otherwise other. |
| ADL (Activity of daily living) | ADL was defined as daily activity including eating, dressing, getting into or out of a bed or chair, taking a bath or shower and using toilet independently. Disturbance is assessed as the requirement of partial or total assistance for these activities before the accidental hypothermia event, based on the judgement of the physicians or nurses in charge or their families. |
| Welfare | Living on public assistance |
| Clinical information on hospital arrival | |
| Cardiac arrest | Cardiac arrest confirmed at ED arrival |
| Vital signs | Body temperature [BT](℃), Systolic blood pressure [SBP] (mmHg), heart rate [HR] (bpm), and Glasgow coma scale [GCS] at ED arrival  BT is a core body temperature measured at rectum, bladder or esophageal if available. If it is not measured, BT is surface BT measured by axillary, tympanic or others. If BT in ED is missing, it is imputed by BT measured in pre-hospital setting. |
| Blood test results | Following blood test results on ED arrival: pH on blood gas assessment, hemoglobin [Hb], (g/dL), Hematocrit [Ht], (%), Platelet count [Plt], (x10^3^/uL), Blood urea nitrogen [BUN], (mg/dL), Creatinine, serum [Cre] (mg/dL), Serum sodium [Na^+^] (mEq/L), Serum potassium [K^+^], (mEq/L), Serum Chloride [Cl^-^] (mEq/L), Serum Calcium [Ca^2+^] (mmol/L), Total calcium [Ca] (mg/dL), |
| Rewarming method | |
| Warm Fluid | Iv warm fluid |
| Blanket | Blanket |
| Forced Warm Air | Forced Warm Air |
| Intravascular Catheter | Intravascular catheter with warm water-circulating balloon catheter |
| HD/CHDF | Hemodialysis or continuous hemodialysis or filtration |
| VA ECMO | Emergency implementation of veno-arterial extracorporeal membrane oxygenation. |
| Other Information | |
| Disposition | The disposition after the initial treatment at ED: Discharge/ Admission to ward/Admission to ICU |
| Mortality | In-hospital mortality |

ED: Emergency department

**eAppendix4. Estimation of the albumin value and sensitivity analysis**

For calculating the probability of 5A score model, serum albumin value on hospital arrival is necessary; however, it is not recorded in the JAAM Hypothermia 2018-2019 database. Thus, we calculated estimated albumin value using J-point registry database by following methods.

The detail of J-point registry is described in some previous reports.[2, 3] In summary, J-point registry database is a multicenter retrospective cohort study collecting clinical information among the accidental hypothermia patients between April 1, 2011, and March 31, 2016, conducted in 12 hospitals in urban areas of the Kyoto, Osaka, and Shiga prefectures in Japan. The ethics committee of each center approved the registry and retrospective analysis of de-identified data (Ethical approval ID of representative institution, Kyoto Prefectural University of Medicine: ERB-C-633).

Of 572 accidental hypothermia patients in this database, 532 adult accidental hypothermia patients whose body temperature was 35℃ or less were selected. Of them, the patients whose total calcium and albumin value were missing or outlier were excluded, finally, 391 patients were included for developing estimating equation. Statistical analyses were performed using R software (version 4.0.3) and statistical package “caret” and “ranger”.

1. Main analysis: Estimation by liner regression with the total calcium value

First, we develop the estimating equation using total calcium value. Generally, serum albumin binds approximately 40-50% of serum total calcium and liner relationship between serum albumin and total calcium has reported.[4-8] The scatter plot is described to indicate the relationship between total calcium and albumin value (See below).

We fitted the liner regression model as follows:

Alb (mg/dL) = 0.6169*Total Ca (mg/dL) -1.9529

Based on the equation, we calculated the estimated albumin value for the patients in JAAM-Hypothermia 2018-2019 database.

1. Sensitivity analysis: Estimation by random forest

For sensitivity analysis, we also developed the estimation model using random forest model to calculate albumin value. Random forest is a kind of machine learning consist of decision tree model with bootstrap samples. We fitted the random forest model using J-point registry data using following predictors: Age, sex, body temperature, pH, Na, K, Cl, Ca, Cre, BUN, Hb, Ht and Plt as mentioned in **eAppendix3**. Using this model, we also calculated estimated albumin value and perform the analysis as same as estimation of liner regression.

The variable importance of the Random Forest model

| Predictors | Importance | Predictors | Importance | Predictors | Importance |
| --- | --- | --- | --- | --- | --- |
| Ca | 100 | BUN | 6.3971 | Sex | 0.2872 |
| Hb | 19.1629 | Na^+^ | 1.5271 | Cre | 0.2258 |
| Ht | 12.0651 | Cl^-^ | 1.3251 | pH | 0 |
| Age | 8.4044 | K^+^ | 1.0958 | - | - |
| Plt | 8.2738 | BT | 0.3186 | - | - |

Abbreviation and definition in each variable are described in **eAppendix3.**

1. Sensitivity analysis: Worst case scenario

Further, we also performed sensitivity analysis assuming the two extreme situations in which the albumin value in all the patients in JAAM-Hypothermia database 2018-2019 were more than 3 mg/dl and lower than 3 mg/dl, to evaluate the influence of albumin value on the main results. The scenario that albumin value of all the patients were assumed more than 3 mg/dl scored means that all the patients were scored 0 point at the albumin predictor, and the other scenario that albumin value of all the patients were assumed lower than 3 mg/dl means that all the patients were scored 1 point at the albumin predictor.

**eAppendix5. Missing of the variables**

Missing or Unknown in each variable is described as number and percentage.

| Characteristic | N = 1,139 |
| --- | --- |
| Age | 0 (0%) |
| Sex | 0 (0%) |
| Route to hospital | 8 (0.7%) |
| Primary cause | 84 (7.4%) |
| Setting | 33 (2.9%) |
| Welfare | 57 (5.0%) |
| ADL | 33 (2.9%) |
| Cardiac arrest on arrival | 25 (2.2%) |
| BT | 0 (0%) |
| SBP | 89 (7.8%) |
| HR | 40 (3.5%) |
| GCS | 127 (11%) |
| pH | 106 (9.3%) |
| Hb | 46 (4.0%) |
| Ht | 55 (4.8%) |
| Plt | 50 (4.4%) |
| BUN | 49 (4.3%) |
| Cre | 51 (4.5%) |
| Na^+^ | 44 (3.9%) |
| K^+^ | 44 (3.9%) |
| Cl^-^ | 45 (4.0%) |
| Ca | 230 (20%) |
| Disposition | 45 (4.0%) |
| Mortality | 100 (8.8%) |
| Abbreviation and definition are described in **eAppendix3.** | |

**eAppendix6. Other detail of patient characteristics**

| Characteristics | N = 1,139 |
| --- | --- |
| Lifestyle |  |
| Homeless | 5 (0.4%) |
| Living Alone | 362 (32%) |
| Nursing Home | 52 (4.6%) |
| BT measurement site |  |
| Axillary | 316 (28%) |
| Bladder | 476 (42%) |
| Esophageal | 43 (3.8%) |
| Other/Unknown | 74 (6.5%) |
| Rectal | 201 (18%) |
| Tympanic | 29 (2.5%) |
| Blood test result |  |
| pH | 7.29 (7.18, 7.35) |
| Hb | 12.2 (10.1, 13.7) |
| Ht | 37 (31, 41) |
| Alb (estimated by liner regression) | 3.54 (3.23, 3.77) |
| Alb (estimated by Random Forest) | 3.57 (3.17, 3.88) |
| Plt | 19 (13, 25) |
| BUN | 31 (19, 53) |
| Cre | 1.08 (0.71, 1.84) |
| Na | 140 (136, 143) |
| K | 4.2 (3.7, 4.9) |
| Cl | 103 (99, 106) |
| Ca | 8.90 (8.40, 9.28) |
| Disposition |  |
| Discharge | 136 (12%) |
| ICU | 610 (54%) |
| Ward | 393 (35%) |
| Year |  |
| 2018-19 | 572 (50%) |
| 2019-20 | 567 (50%) |

Abbreviation and definition are described in **eAppendix3**. Detail of Albumin value estimation is explained in **eAppendix3**. Continuous variables are described as median and interquartile range and categorical variables are number and percentage.

**eAppendix7. Performance of 5A score model**

| Parameter | Value |
| --- | --- |
| **R^2^** | 0.196 |
| **Brier** | 0.151 |
| **Intercept** | -0.008 |
| **Slope** | 0.836 |

**eAppendix8. Predicted and Observed probability**

| Sum Score | Number of Mortality | N | Observed  Probability | Predicted Probability | 95%CI of Predicted probability | |
| --- | --- | --- | --- | --- | --- | --- |
| 0 | 1 | 26 | 0.038 | 0.014 | 0.024 | 0.004 |
| 1 | 5 | 87 | 0.057 | 0.03 | 0.041 | 0.019 |
| 2 | 22 | 151 | 0.146 | 0.055 | 0.069 | 0.041 |
| 3 | 32 | 219 | 0.146 | 0.103 | 0.119 | 0.087 |
| 4 | 59 | 334 | 0.177 | 0.191 | 0.208 | 0.174 |
| 5 | 63 | 193 | 0.326 | 0.332 | 0.365 | 0.299 |
| 6 | 38 | 66 | 0.576 | 0.524 | 0.583 | 0.465 |
| 7 | 39 | 49 | 0.796 | 0.681 | 0.732 | 0.63 |
| 8 | 10 | 12 | 0.833 | 0.84 | 0.921 | 0.759 |
| 9 | 2 | 2 | 1.000 | 0.927 | 0.927 | 0.927 |

CI: Confidence Interval

**eAppendix9. Results of Sensitivity Analysis using estimation by random forest**

Sensitivity analysis: Albumin value estimated by Random forest model

Albumin value is estimated by random forest model using J-point data (see **eAppendix5.**). The results are almost same as the main analysis.

**eAppendix10. Results of Sensitivity Analysis usin**g **worst case scenario**

The detail of sensitivity analysis is described in **eAppendix5.**

Red: Assumption that albumin value in all the patients are less than 3mg/dl.

Blue: Assumption that albumin value in all the patients are more than 3mg/dl.

These assumptions indicate extreme situations of albumin value. Accordingly, even if the albumin value was not estimated accurately, the calibration plot would be expected to exist within the range between blue and red lines, and it indicates the robustness that the 5A score is well calibrated.

**Reference**

1. Okada Y, Matsuyama T, Morita S, Ehara N, Miyamae N, Jo T, Sumida Y, Okada N, Watanabe M, Nozawa M *et al*: **The development and validation of a "5A" severity scale for predicting in-hospital mortality after accidental hypothermia from J-point registry data**. *J Intensive Care* 2019, **7**:27.

2. Matsuyama T, Morita S, Ehara N, Miyamae N, Okada Y, Jo T, Sumida Y, Okada N, Watanabe M, Nozawa M *et al*: **Characteristics and outcomes of accidental hypothermia in Japan: the J-Point registry**. *Emerg Med J* 2018, **35**(11):659-666.

3. Okada Y, Matsuyama T, Morita S, Ehara N, Miyamae N, Jo T, Sumida Y, Okada N, Kitamura T, Iiduka R: **Prognostic factors for patients with accidental hypothermia: a multi-institutional retrospective cohort study**. *The American journal of emergency medicine* 2019, **37**(4):565-570.

4. Thambiah S-C-: **Derivation and Internal Validation of an Equation for Albumin-adjusted Calcium at a Tertiary Hospital in Selangor, Malaysia**. *Malaysian Journal of Medicine and Health Sciences* 2020:16-25.

5. James MT, Zhang J, Lyon AW, Hemmelgarn BR: **Derivation and internal validation of an equation for albumin-adjusted calcium**. *BMC clinical pathology* 2008, **8**:12-12.

6. Davies SL, Hill C, Bailey LM, Davison AS, Milan AM: **The impact of calcium assay change on a local adjusted calcium equation**. *Annals of Clinical Biochemistry* 2015, **53**(2):292-294.

7. Pawade YR, Ghangale SS, Dahake HS: **Albumin-adjusted calcium: Are previously published regression equations reliable for your laboratory?-A pilot study**. 2013.

8. Ariffin ZAM, Jamaluddin FA: **Albumin adjusted calcium: Study in a tertiary care hospital**. *The Malaysian journal of pathology* 2020, **42**(3):395-400.
